# Validity of self-reported measures of decreased muscle strength in older adults

**DOI:** 10.1590/0034-7167-2024-0232

**Published:** 2025-10-03

**Authors:** João Paulo Neves Mota, Cristina Camargo Pereira, Milara Barp, Daniella Pires Nunes, Cynthia Assis de Barros Nunes, Maria Márcia Bachion, Erika Aparecida Silveira, Valéria Pagotto

**Affiliations:** I Universidade Federal de Goiás. Goiânia, Goiás, Brazil; II Universidade Estadual de Campinas. Campinas, São Paulo, Brazil

**Keywords:** Geriatric Assessment, Aging, Muscle Strength, Health of the Elderly, Sarcopenia, Evaluación Geriátrica, Envejecimiento, Fuerza Muscular, Salud del Anciano, Sarcopenia

## Abstract

**Objectives:**

to analyze the validity of self-reported measures for screening decreased muscle strength in older adults.

**Methods:**

this cross-sectional study assessed muscle strength using the gold-standard Handgrip Strength (HGS) test and the application of four subjective measures: A = perceived reduction in strength; B = difficulty lifting 5 kg; C = low score on the SARC-F; and D = combination of measures A + B. Validity was determined through sensitivity, specificity, positive predictive value (PPV), negative predictive value (NPV), and accuracy, with a 95% confidence interval (95% CI).

**Results:**

a total of 18.7% of participants exhibited decreased muscle strength, while subjective measures showed a frequency variation between 74.2% and 35.5%. Measure A demonstrated the highest sensitivity, whereas Measure C exhibited the highest specificity. The accuracy of Measures A, B, C, and D was 61.5%, 41.2%, 34.8%, and 36.2%, respectively

**Conclusions:**

self-reported measures demonstrated low accuracy; however, Measures A and C, due to their ease of application, could be used in combination.

## RESUMEN

**Objetivos:**

analizar la validez de medidas autoinformadas para la detección de la fuerza muscular reducida en personas mayores.

**Métodos:**

estudio transversal que evaluó la fuerza muscular mediante la medida estándar de referencia Fuerza de Prensión Palmar (FPP) y la aplicación de cuatro medidas subjetivas: A = reducción de la fuerza; B = dificultad para levantar 5 kg; C = baja puntuación en el SARC-F; y D = combinación de las medidas A + B. La validez se determinó mediante sensibilidad, especificidad, valores predictivos positivo (VPP) y negativo (VPN) y precisión, con un Intervalo de Confianza del 95% (IC95%).

**Resultados:**

el 18,7% presentó fuerza reducida; las medidas subjetivas mostraron una variación en la frecuencia entre 74,2% y 35,5%. La medida A presentó la mayor sensibilidad, mientras que la medida C mostró la mayor especificidad. La precisión de las medidas A, B, C y D fue respectivamente de 61,5%, 41,2%, 34,8% y 36,2%.

**Conclusiones:**

hubo baja precisión en las medidas autoinformadas; sin embargo, las medidas A y C presentan fácil aplicabilidad y podrían utilizarse en combinación.

## INTRODUCTION

Reduction in muscle strength is a common condition in older adults and occurs due to physiological changes resulting from aging^(1)^, as well as conditions and factors accumulated throughout life, such as diabetes mellitus^(2)^, lower body mass index (BMI), and higher waist circumference (WC)^(3)^.

Studies show that, starting at the age of 50, there is a decline in muscle mass (1% to 2% per year) and muscle strength (1.5% to 5% per year)^(4)^. These phenomena can impact the performance of daily living activities^(5)^, increase depressive symptoms^(6)^, and lead to hospitalizations and death^(7)^. Due to its clinical significance, reduced muscle strength is a symptomatic component of two important and current geriatric syndromes: frailty^(8)^ and sarcopenia^(4)^, both correlated with dependence and loss of autonomy.

Data from the Longitudinal Study of Health of Older Brazilians (ELSI-Brazil), conducted from 2015 to 2016, showed that nearly one-fifth of Brazilian older adults were classified as having reduced muscle strength. Additionally, it was found that 10.0% of cases of reduced muscle strength could be attributed to modifiable risk factors, such as physical inactivity, and 16.0% to lower educational attainment^(9)^. In the context of sarcopenia, it is estimated that the prevalence of reduced muscle strength affects 38.3% of individuals aged 70 years or older^(10)^.

Despite the clinical and epidemiological importance of muscle strength for the quality of life of older adults and its long-term impact on care provided by both families and healthcare services^(5)^, its assessment remains a challenge in routine healthcare settings^(11)^.

Handgrip strength (HGS) is a test used to assess the muscle capacity of the upper limbs to grasp an object, serving as an indicator of overall muscle strength and being measured using a dynamometer^(12)^.

However, although measurement devices such as dynamometers are relatively low-cost and easy to use, their application remains underutilized in clinical practice^(11)^. Professionals encounter considerable variability in testing protocols, different types of equipment used for assessments, and a wide range of implementation procedures and reference values. These inconsistencies can lead to the inconsistent adoption of this assessment in clinical practice^(11,12)^.

Given this, the use of self-reported measures may be an alternative for integrating muscle strength assessment into routine healthcare for older adults. However, these measures must demonstrate validity. Thus, analyzing the use of self-reported muscle strength measures is essential to enable their application in healthcare services, considering the impact of this variable on the health of older adults.

In Brazil, the validity of self-reported muscle strength was first demonstrated in the Health, Well-being, and Aging Study (SABE in Portuguese)^(13)^. In this study, self-reported muscle strength, obtained through self-reported muscle strength loss over the previous 12 months, exhibited high sensitivity, good factorial load, and internal consistency. It was recommended as a valid tool for assessing one of the fundamental elements of the self-reported frailty construct^(13)^. However, following a review of the cutoff points proposed in the 2018 sarcopenia consensus^(4)^ for reduced muscle strength and with the introduction of the Simple Questionnaire to Rapidly Diagnose Sarcopenia (SARC-F)^(14)^, which includes other subjective strength measures for sarcopenia screening in older adults, further evaluation of these new parameters is necessary.

## OBJECTIVES

To analyze the validity of self-reported muscle strength measures in older adults, both overall and by sex.

## METHODS

### Ethical aspects

The study was conducted in accordance with national and international ethical guidelines and was approved by the Research Ethics Committee of the Clinical Hospital of the Federal University of Goiás (CEP/FC/UFG), whose approval document is attached to this submission. Written informed consent was obtained from all individuals involved in the study.

### Study design, period, and location

This cross-sectional study used data from the cohort “*Projeto Idosos/Goiânia*”, conducted between 2008 and 2018. The study cohort consisted of a representative population of community-dwelling older adults, users of the Brazilian Unified Health System (SUS in Portuguese), aged ≥60 years, residing in the city of Goiânia, Goiás, in the Central-West region of Brazil. This population was estimated proportionally to the number of older adults living in the nine health districts of Goiânia.

The study’s sampling procedures, previously described in earlier research ^(15,16)^, were carried out in multiple stages:

1. The total sample size was proportionally allocated among the nine municipal health districts (HD), considering the proportion of older adults residing in each district.
2. In these units, the number of older adults who had received care in the previous 12 months was listed.
3. Based on this listing, a simple random allocation was performed to select the participants^(15,16)^.

The STROBE instrument was used to guide the preparation of this article^(17)^.

### Population, inclusion, and exclusion criteria

The study included 187 older adults with complete data on both physical and self-reported HGS, along with other covariates described in the study protocol. Individuals who exhibited cognitive decline, as assessed by the Mini-Mental State Examination (MMSE)^(18)^, were excluded.

### Study Protocol

Interviews and measurements were conducted by previously trained researchers following standardized procedures.

The primary variable in this study was decreased muscle strength, assessed through both self-reported and measured evaluations (gold standard)^(19)^.

For the assessment of self-reported muscle strength, four parameters, referred to as Measures A, B, C, and D, were used:

- Measure A. Perceived reduction in strength: assessed by a positive response to the question, “In the past 12 months (last year), do you feel weaker? Do you think your strength has decreased?”^(19)^.
- Measure B. Self-reported difficulty in lifting 5 kg: assessed by a positive response (some, a lot, or no difficulty) to the SARC-F instrument question^(14)^: “How much difficulty do you have lifting and carrying 5 kg?”.
- Measure C. Low SARC-F score: defined as a total score of ≥4 on the SARC-F instrument, which predicts sarcopenia^(14)^.
- Measure D. Perceived reduction in strength + self-reported difficulty in lifting 5 kg: refers to the combination of parameters A and B, where older adults who answered “yes” to both questions were considered to have low strength.

Regarding the gold standard parameter, HGS was measured using a Jamar^®^ hydraulic dynamometer. For the test, participants were instructed to sit in a chair without armrests, flex their elbows, and then press the device handle with the palm of their hand, applying maximum force. The test was performed three times on each arm, alternating sides with rest intervals between attempts. For analysis, the highest HGS value obtained was considered.

Muscle strength was dichotomized as normal or decreased. Decreased muscle strength was defined according to the cutoff points recommended by the European Working Group on Sarcopenia in Older People (EWGSOP2), with <27 kgf for men and <16 kgf for women^(4)^.

Independent variables included age, sex, number of comorbidities, and BMI. Weight was measured using a digital electronic scale (Tanita^®^) with a precision of 0.01 grams. Height was measured using a portable stadiometer. The presence of comorbidities was assessed based on the participant’s self-report of medical diagnoses, using the question: “Which diseases have you been diagnosed with by a doctor?”. A list of 17 diseases was considered: diabetes, hypertension, overweight/obesity, malnutrition, high cholesterol, high triglycerides, osteoporosis, cancer, stroke, myocardial infarction, asthma, bronchitis, Chronic Obstructive Pulmonary Disease (COPD), cataracts, migraines, depression, and gastritis.

### Analysis of results and statistics

Data were analyzed using STATA software, version 14.0.

Initially, all variables were analyzed descriptively using the mean, median, standard deviation, absolute frequency, and relative frequency. Subsequently, the sample was characterized based on the mean and standard deviation (SD).

The validity of self-reported muscle strength (Measures A, B, C, and D) was analyzed in relation to the gold standard method (HGS)^(4,20,21)^. Validity was determined based on five characteristics: sensitivity, specificity, positive predictive value (PPV), negative predictive value (NPV), and accuracy.

To achieve this, a 2×2 table was constructed, comparing the gold standard with each self-reported measure, allowing for the calculation of the following validity measures:

1. Sensitivity: the proportion of true positives (positive according to both self-reported and measured evaluations) divided by the sum of true positives and false negatives (positive by measured evaluation but negative by self-reported evaluation).
2. Specificity: the proportion of true negatives (negative according to both self-reported and measured evaluations) divided by the sum of true negatives and false positives (negative by measured evaluation but positive by self-reported evaluation).
3. PPV: the proportion of true positives divided by the sum of true positives and false positives.
4. NPV: the proportion of true negatives divided by the sum of true negatives and false negatives.
5. Accuracy: the proportion of the sum of true positives and true negatives relative to the total sample (N).

## RESULTS

The sample consisted of 187 community-dwelling older adults, of whom 66.8% were women, with a mean age of 78.8±5.9 years. Participants had an average of 4.0±2.1 comorbidities and a BMI of 27.3±5.3 kg/m^2^. Women had lower HGS (20.4±5.5 kg) compared to men (32.7±9.4 kg) (p<0.001) (Table 1).

**Table 1:** Characteristics of the total sample and by sex, Goiânia, Goiás, Brazil, 2018

Variables	Total (N = 187)		Women (n = 125)		Man (n = 62)		*p* value
	Mean	±SD	Mean	±SD	Mean	±SD	
Mean	78.8	5.9	78.7	6.1	79.1	5.4	0.092
Age (years)	4.0	2.1	4.1	2.2	3.8	2.1	0.294
Multimorbidity	27.3	5.3	27.7	5.6	26.5	4.5	0.466
BMI (kg/m^2^)	24.5	9.1	20.4	5.5	32.7	9.4	**<0.001**

*SD - Standard Deviation; HGS - Handgrip Strength; BMI - Body Mass Index.*

**Figure 1:**
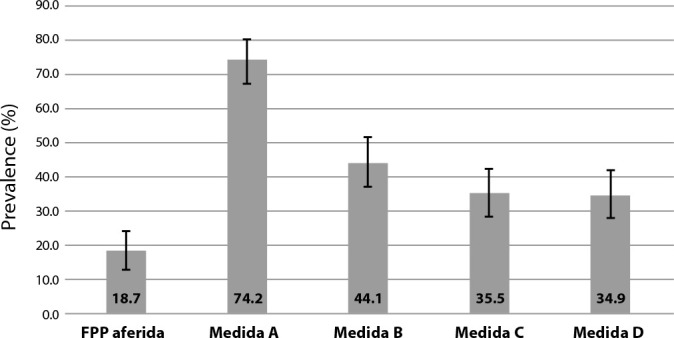
Prevalence of decreased muscle strength in older adults according to measured and self-reported criteria, Goiânia, Goiás, Brazil, 2018

*Note: HGS: Handgrip Strength (measured and classified according to the EWGSOP cutoff point); Measures A, B, C, and D (self-reported, based on different criteria cited in the methodology).*

The prevalence of decreased muscle strength in older adults varied according to the assessment method (Figure 1), with the lowest prevalence observed when using HGS as the reference.

Regarding the predictive capacity of the four self-reported strength measures, Table 2 shows that perceived strength reduction (Measure A) exhibited the highest sensitivity, PPV, and accuracy among the variables analyzed, with similar values between women and men. A low SARC-F score (Measure C) demonstrated the highest specificity, particularly among men; however, it had lower accuracy compared to the other measures evaluated. It is noteworthy that all subjective methods assessed showed significant PPV, except for Measure C in men.

**Table 2:** Predictive capacity of self-reported muscle strength measures in relation to the gold standard, Goiânia, Goiás, Brazil, 2018

Subjective Strength Measures	Handgrip Strength (Gold Standard)				
	Sensitivity (IC 95%)	Specificity (IC 95%)	PPV (%)	NPV (%)	Accuracy (%)
Overall					
Measure A	71.7	17.1	79.0	12.2	61.5
Measure B	41.1	41.7	74.7	14.4	41.2
Measure C	31.7	74.2	71.6	14.2	34.8
Measure D	32.5	52.9	75.4	15.0	36.2
Women					
Measure A	71.7	16.7	83.5	9.1	63.7
Measure B	47.2	21.1	76.9	6.7	43.2
Measure C	38.7	31.6	76.9	8.5	37.6
Measure D	36.8	38.9	78.0	9.5	36.8
Men					
Measure A	71.7	12.5	70.2	13.3	56.5
Measure B	26.7	68.8	70.6	25	37.7
Measure C	15.5	68.8	58.3	22.4	29.5
Measure D	22.2	68.8	66.7	23.9	34.4

*A - perceived reduction in strength; B - difficulty lifting 5 kg; C - low SARC-F score; D - reduction in strength + lifting 5 kg; CI - Confidence Interval; PPV - Positive Predictive Value; NPV - Negative Predictive Value.*

## DISCUSSION

Seeking to contribute to the planning of sustainable actions in the healthcare system that meet the needs of older adults, this study analyzed the validity of self-reported measures used in research to assess decreased muscle strength, considering HGS as the gold standard. The goal was to identify evaluations with high sensitivity and good specificity. Overall, the results showed that the investigated Measures A and C adequately met one or the other parameter but not both simultaneously, making them ineligible for confirming the studied phenomenon.

However, considering other properties such as accuracy and positive predictive value, Measure A demonstrated good accuracy and positive predictive value for women (83.5% and 63.7%, respectively). For men, none of the measures showed adequate accuracy. This scenario suggests that, in the absence of a dynamometer for evaluating HGS, other measures should be used in combination, especially for men, to support decision-making regarding necessary interventions by the multidisciplinary team, in which nursing can play a significant role.

Additionally, it is important to highlight that in studies where decreased muscle strength is a variable of exposure or outcome, self-reported measures should be avoided.

In the present study, considering the result obtained using a hydraulic dynamometer (the adopted gold standard measure), the prevalence of low muscle strength found (18.7%) was higher than that observed in the cohort of older adults in the SABE study^(22)^, which reported 7.5%. However, it was similar to the results of the ELSI cohort, which, using the same cutoff points, found a prevalence of 16.6% among men and 17.7% among women^(9)^. These discrepancies are due to the different cutoff points adopted in the mentioned studies. Both this investigation and the ELSI-Brazil study used the cutoff points recommended by EWGSOP2, a widely adopted parameter in international studies, where similar prevalence rates to those found in this study have been reported^(23,24)^.

Low muscle strength estimated by Measure A (low perceived strength) showed the highest prevalence (74.2%) compared to the other measures. Results from studies that used this form of muscle strength assessment show wide variation, such as 80.0% in Minas Gerais^(25)^ and 40.7% in Tocantins^(26)^.

Measure B (difficulty lifting 5 kg), reported by 44.7% of older adults in this study, was previously cited by 27.3% of Korean older adults^(27)^ and by 35% of older adults in Great Britain^(28)^.

Measure C (score ≥4 on the SARC-F), which indicated a prevalence of 35.5% for low muscle strength, has been reported in other studies with a prevalence of 18.8% in Turkish older adults over 65 years old^(29)^, 16.6% in Chinese older adults over 65 years old^(30,31)^, and 39.8% in Chinese older adults over 70 years old^(30,31)^. In Brazil, the estimated prevalence based on Measure C varied widely, ranging from 4.5% in women over 60 years old in the Southern Region^(32)^ to 23.0% in older adults of both sexes in the Southeastern Region^(33)^.

The differences in prevalence among self-reported measures may be attributed to their semantic construction. Subjective measures are known to be susceptible to bias, as responses depend on how individuals interpret the questions. For instance, the question related to Measure A (perceived reduction in strength over the past 12 months) is more general and, therefore, may reflect overall strength. Additionally, it refers to a dynamic state of change, meaning that respondents assess the reduction in strength rather than whether it has actually fallen below the optimal level for functionality.

Measures B and C, by including the criterion “difficulty carrying more than 5 kg”, may be linked to a more specific perception. This could directly impact the number of cases identified as positive. It is important to consider that when establishing comparative standards, such as lifting and carrying 5 kg, it would be advisable to refine the wording of the question to enhance construct clarity. A possible alternative would be: “Difficulty carrying a 5 kg bag of rice for 30 seconds?”. The 30-second duration aligns with the cutoff point for the gait speed test^(4)^. This modification could improve the definition of the assessment scenario and reduce varied interpretations that might compromise comparative measures within the same population.

It is also worth noting that these two measures have been presented with only two response options. For future studies, it is recommended to include response options such as “none”, “mild”, “moderate”, and “severe” to determine whether different cutoff points provide better sensitivity, specificity, NPV, PPV, and accuracy.

The instrument proposed by Nunes et al.^(19)^ was originally developed for detecting frailty syndrome in older adults based on the frailty phenotype^(8)^. In that context, the strength-related item demonstrated good sensitivity (77.7%) for frailty but, on the other hand, had a specificity of 34.9%, a PPV of 44.7%, and an NPV of 69.8%^(19)^. Since the strength loss item of the self-reported instrument showed a good factor loading and internal consistency^(19)^, it was considered a potential tool for the exclusive assessment of strength reduction. This measure has a high capacity to identify true positives, and although it does not effectively exclude true negatives, it remains a promising tool for screening older adults, enabling future evaluations and interventions related to strength reduction^(34,35)^.

Measure B showed good PPV values in both the overall sample and the sex-stratified analysis, in addition to a specificity of 68.8% for men. No other studies were found that specifically demonstrated the sensitivity, specificity, PPV, NPV, and accuracy of the strength-related item in the SARC-F. This is likely due to the use of a more specific comparison standard, which allows older adults to assess whether they meet this criterion, as previously mentioned.

Regarding Measure C (low SARC-F score), international studies have reported variations in sensitivity values, ranging from 15% to 40.3%, and specificity values ranging from 82% to 99% for both sexes^(27-29,36)^.

In the Southern Region of Brazil, Measure C showed a sensitivity of 58.9% and a specificity of 82.1%^(14)^. Similar values were observed in the Southeastern Region, with a sensitivity of 60.0% and a specificity of 80.92%^(37)^.

When the sample is stratified by sex, a significant decrease in the specificity of Measure C (low SARC-F score) is observed among women. Similar results were found in a Chinese study^(38)^ and a Korean study^(27)^. No studies of this nature were identified in Brazil.

Due to the numerous complications associated with reduced muscle strength, such as increased mortality, hospitalization, falls, and decreased independence and autonomy in older adults^(5-7)^, early detection of this condition is essential to guide health interventions, particularly in the context of primary care^(19,39)^.

### Study limitations

The study included participants who were users of the primary healthcare network, which may be a limiting factor for the reproducibility of the estimated prevalence of decreased muscle strength in the general older adult population.

Another limitation concerns potential confounding factors that were not included in the validity analysis of these measures, such as acute, inflammatory, and musculoskeletal conditions, which may affect the measurement of muscle strength in older adults and, consequently, impact the validity of the measures. Despite this, multimorbidity among older adults was analyzed using a list of 17 chronic conditions, which are common and recommended for use in studies involving this population.

However, considering the primary objective of this investigation, which was to estimate the sensitivity, specificity, positive and negative predictive values, and accuracy of self-reported muscle strength measures, the study’s findings remain relevant.

### Contributions to nursing and public health

Considering the goal of promoting healthy aging among the older population and optimizing functional capacities over the years, incorporating muscle strength monitoring by nurses in PHC enables the early identification of functional changes^(39)^, supporting preventive and rehabilitative actions for older adults receiving care in PHC.

In this context, this study demonstrated that self-reported Measures A (perceived reduction in muscle strength) and C (low SARC-F score) are useful for integration into clinical nursing practice and can be incorporated into nursing consultations for comprehensive geriatric assessment (CGA).

Additionally, when included in routine assessments, alongside the evaluation of vital signs and BMI, the use of a self-reported test for muscle strength assessment can serve as a screening tool for sarcopenia and frailty, offering ease of application and low cost.

Muscle strength assessment also provides an opportunity for health education on strategies to preserve muscle function, such as regular physical activity, particularly strength training exercises. Furthermore, it enables guidance on fall risks for older adults and their caregivers.

It is important to note that although the focus of this investigation is the older population, reduced muscle strength is a related factor (causal/contributory factor) or a defining characteristic (evidence) in fourteen different nursing diagnoses in the NANDA-I taxonomy^(40)^. This underscores the applicability of the study’s findings in various practice settings and highlights the need for future studies to validate nursing diagnoses.

Considering that the question about “lifting and carrying 5 kg” is included in the *Caderneta de Saúde da Pessoa Idosa* (Older Adult Health Record) proposed by the Ministry of Health^(41)^, it is essential to emphasize that the findings of this investigation serve as a caution for healthcare professionals. This item should not be used as the sole parameter for assessing the presence of reduced muscle strength. Thus, the study contributes to evidence-based practice in primary healthcare services for older adults regarding muscle strength assessment.

The promotion of healthy aging in the older population involves optimizing functional abilities throughout life. In this regard, muscle strength is a key indicator of mobility changes and should be monitored in the context of PHC^(39)^.

Assessing muscle strength in older adults within nursing practice helps in the early identification of strength decline, a condition associated with issues such as frailty, falls, loss of independence, and increased risk of hospitalization.

This study demonstrated that self-reported Measures A (perceived reduction in muscle strength) and C (low SARC-F score) are valuable for integration into clinical nursing practice, whether in nursing consultations, physical examinations of older adults, or screening campaigns. Additionally, these measures can support the planning of specific or interdisciplinary care interventions, involving family participation in the short, medium, and long term, as they are easy-to-apply technologies.

It is important to highlight that, although the focus of this investigation is the older population, reduced muscle strength is identified as a related factor (causal/contributory factor) or a defining characteristic (evidence) in fourteen different nursing diagnoses within the NANDA-I taxonomy^(40)^. This underscores the applicability of the study’s findings in various clinical settings and emphasizes the need for future studies to validate nursing diagnoses.

Considering that the question about “lifting and carrying 5 kg” is included in the *Caderneta de Saúde da Pessoa Idosa* (Older Adult Health Record) proposed by the Ministry of Health^(41)^, it is essential to stress that the findings of this investigation should serve as a caution for healthcare professionals. This item should not be used as the sole parameter for assessing the presence of reduced muscle strength. Thus, this study contributes to evidence-based practice among healthcare professionals in primary care services for older adults, particularly regarding muscle strength assessment.

## CONCLUSIONS

For a reliable diagnosis and an adequate predictive value of decreased muscle strength, the use of valid measures, such as HGS, is necessary, along with alternative methods when a dynamometer is unavailable. In this regard, Measures A and C appear to be the most suitable for screening muscle strength loss among older adults in PHC.

Measure A, due to its high capacity for identifying individuals for screening, and Measure C, due to its ability to exclude false positives, may together indicate a priority group for intervention. However, these measures were not sufficiently adequate to be recommended as standalone screening tools for detecting muscle strength reduction among community-dwelling older adults in healthcare services. Nevertheless, considering their ease of application in clinical and professional practice, adopting these parameters may be useful for identifying older adults who may require preventive interventions to mitigate adverse health outcomes associated with muscle strength loss.

## Data Availability

The research data are available within the article.
