# Supplementary material for: Validity of self-reported measures of decreased muscle strength in older adults
Source: Rev Bras Enferm. 2025 Oct 3;78(Suppl 2):e20240232. doi: 10.1590/0034-7167-2024-0232 (PMC12500073; doi:10.1590/0034-7167-2024-0232)
Supplement: Translation

# Translation: Validade de medidas autorreferidas de força muscular diminuída em idosos

**DOI:** 10.1590/0034-7167-2024-0232pt

**Authors:** João Paulo Neves Mota, Cristina Camargo Pereira, Milara Barp, Daniella Pires Nunes, Cynthia Assis de Barros Nunes, Maria Márcia Bachion, Erika Aparecida Silveira, Valéria Pagotto

**Affiliations:** I Universidade Federal de Goiás. Goiânia, Goiás, Brasil; II Universidade Estadual de Campinas. Campinas, São Paulo, Brasil

**Keywords:** Avaliação Geriátrica, Envelhecimento, Força Muscular, Saúde do Idoso, Sarcopenia

## Abstract

**Objetivos:**

analisar a validade de medidas autorreferidas para rastreio da força muscular diminuída em idosos.

**Métodos:**

estudo transversal que avaliou a força muscular pela medida padrão ouro Força de Preensão Palmar (FPP) e aplicação de quatro medidas subjetivas: A= redução da força; B = dificuldade de levantar 5kg; C = baixa pontuação no SARC-F e D = medidas A+B. A validade foi determinada por meio da sensibilidade, especificidade, valores preditivos positivo (VPP) e negativo (VPN) e acurácia, com Intervalo de Confiança de 95% (IC95%).

**Resultados:**

18,7% apresentaram força diminuída, medidas subjetivas tiveram variação de frequência entre 74,2% e 35,5%. A medida A apresentou maior sensibilidade e a medida C maior especificidade. A acurácia das medidas A, B, C e D foi respectivamente de 61,5%, 41,2%, 34,8% e 36,2%.

**Conclusões:**

houve baixa acurácia das medidas autorreferidas, porém, as medidas A e C tem fácil aplicabilidade, poderiam ser utilizadas em associação.

## INTRODUÇÃO

A redução da força muscular é uma alteração comum em idosos e ocorre em função das alterações fisiológicas decorrentes do envelhecimento^(1)^, bem como de condições e fatores acumulados ao longo da vida, tais como diabetes mellitus^(2)^, menor Índice de Massa Corporal (IMC) e maior Circunferência da Cintura (CC)^(3)^.

Estudos mostram que, a partir dos 50 anos, há redução da massa muscular (1 a 2% ao ano) e da força muscular (1,5 a 5% ao ano)^(4)^. Esses fenômenos podem impactar a execução de atividades de vida diária^(5)^, aumentar os sintomas depressivos^(6)^, além de resultar em hospitalizações e óbito^(7)^. Em função de sua importância para a prática clínica, a redução da força muscular é um componente sintomático de duas importantes e atuais síndromes geriátricas: a fragilidade^(8)^ e a sarcopenia^(4)^, ambas correlacionadas à dependência e à perda de autonomia.

Dados do Estudo Longitudinal de Saúde de Idosos Brasileiros (ELSI-Brasil), realizado de 2015 a 2016, mostraram que quase um quinto dos idosos brasileiros foi classificado com força muscular reduzida. Além disso, foi evidenciado que 10,0% da ocorrência da redução da força muscular pode ser atribuída a fatores de risco modificáveis, como o sedentarismo, e 16,0% à menor escolaridade^(9)^. No contexto da sarcopenia, estima-se que a prevalência da redução da força muscular afete 38,3% dos idosos com 70 anos ou mais^(10)^.

Apesar da importância clínico-epidemiológica da força muscular para a qualidade de vida dos idosos e de seu impacto a longo prazo nos cuidados prestados tanto pela família quanto pelos serviços de saúde^(5)^, sua aferição ainda é um desafio na rotina dos serviços de saúde^(11)^.

A força de preensão palmar (FPP) é um teste que permite avaliar a capacidade muscular dos membros superiores para envolver um objeto, sendo um indicador da força muscular do organismo, aplicado por meio de um dinamômetro^(12)^.

Entretanto, embora os equipamentos de aferição, como o dinamômetro, sejam relativamente de baixo custo e fácil aplicação, seu uso ainda é pouco explorado na prática clínica^(11)^. Os profissionais se deparam com uma ampla variabilidade de protocolos, com a indicação de equipamentos de diferentes tipos para a execução dos testes, além de uma grande variação nos procedimentos de realização e nos valores de referência, o que pode levar à incorporação dessa avaliação de modo inconsistente na prática clínica^(11,12)^.

Diante disso, o uso de medidas autorreferidas pode ser uma alternativa para que os cenários de assistência à saúde as incluam na rotina para a mensuração da força muscular em idosos. Para tanto, essas medidas devem apresentar validade. Dessa forma, a análise do uso das medidas autorreferidas de força é importante para permitir sua aplicabilidade nos serviços, dado o impacto dessa variável na saúde dos idosos.

No Brasil, a validade da força muscular autorreferida foi demonstrada primeiramente no estudo Saúde, Bem-estar e Envelhecimento (SABE)^(13)^. Neste estudo, a força muscular autorreferida, obtida por meio do autorrelato da perda de força muscular nos últimos 12 meses, apresentou alta sensibilidade, boa carga fatorial e consistência interna, sendo recomendada para avaliar um dos elementos fundamentais do construto de fragilidade referida^(13)^. No entanto, após a revisão dos pontos de corte propostos no consenso de sarcopenia publicado em 2018^(4)^ para força muscular reduzida, e com a proposta do instrumento *Simple Questionnaire to Rapidly Diagnose Sarcopenia* (SARC-F)^(14)^, que inclui outras medidas subjetivas de força para o rastreamento da sarcopenia em idosos, há necessidade de avaliar os novos parâmetros.

## OBJETIVOS

Analisar a validade da medida autorreferida da força muscular em idosos, de forma geral e segundo o sexo.

## MÉTODOS

### Aspectos éticos

O estudo foi conduzido de acordo com as diretrizes éticas nacionais e internacionais e aprovado pelo Comitê de Ética em Pesquisa do Hospital das Clínicas da Universidade Federal de Goiás (CEP/FC/UFG), cujo parecer está anexado à presente submissão. O Consentimento Livre e Esclarecido foi obtido por escrito de todos os indivíduos envolvidos no estudo.

### Desenho, período e local do estudo

Trata-se de um estudo transversal com dados da coorte “Projeto Idosos/Goiânia”, realizada entre 2008 e 2018. A coorte do estudo foi composta por uma população representativa de idosos da comunidade, usuários do Sistema Único de Saúde (SUS), com idade ≥60 anos, residentes na cidade de Goiânia, Goiás, na região Centro-Oeste do Brasil. Essa população foi estimada de forma proporcional ao número de idosos residentes nos nove distritos de saúde de Goiânia.

Os procedimentos amostrais do estudo, já descritos em pesquisas anteriores^(15,16)^, foram realizados em múltiplos estágios:

1. O número total da amostra foi alocado proporcionalmente entre os nove distritos sanitários (DS) do município, considerando a proporção de idosos residentes em cada DS.
2. Nessas unidades, listou-se a quantidade de idosos atendidos nos 12 meses anteriores.
3. Com base nessa listagem, realizou-se uma alocação aleatória simples para seleção dos participantes^(15,16)^.

Para nortear a elaboração deste artigo, foi utilizado o instrumento STROBE^(17)^.

### População, critérios de inclusão e exclusão

Participaram do estudo 187 idosos que apresentavam dados completos de medidas físicas e autorreferidas referentes à FPP, além das demais covariáveis descritas no protocolo do estudo. Foram excluídos aqueles que apresentaram declínio cognitivo, conforme avaliação pelo Mini Exame do Estado Mental (MEEM)^(18)^.

### Protocolo do estudo

As entrevistas e medições foram realizadas por pesquisadores previamente treinados, seguindo procedimentos padronizados.

A variável principal deste estudo foi a força muscular diminuída, avaliada de forma autorreferida e aferida (padrão-ouro)^(19)^.

Para avaliação da força autorreferida foram utilizados quatro parâmetros, denominadas medidas A, B, C e D:

- Medida A. Percepção de redução de força: mensurada pela resposta positiva à questão “*Nos últimos 12 meses (último ano), o (a) Sr.(a) se sente mais enfraquecido, acha que sua força diminuiu?”* ^(19)^.
- Medida B. Dificuldade referida de levantar 5kg: mensurada pela resposta positiva (alguma, muita ou não tem dificuldade) à questão do instrumento SARC-F^(14)^: *“O quanto de dificuldade o (a) Sr.(a) tem para levantar e carregar 5Kg?”.*
- Medida C. Baixa pontuação no SARC-F: Pontuação ≥4 do escore total do instrumento SARC-F, que prediz sarcopenia^(14)^.
- Medida D. Percepção de redução de força + dificuldade referida de levantar 5kg: Referente a associação dos parâmetros A e B, sendo que foram considerados com baixa força os idosos que responderam sim para as duas questões.

Em relação ao parâmetro padrão-ouro, foi utilizada a FPP, medida com o dinamômetro hidráulico Jamar^®^. Para a realização do teste, o idoso era instruído a se sentar em uma cadeira sem apoio para os braços, flexionar os cotovelos e, em seguida, pressionar a alça do aparelho com a palma da mão, aplicando a maior força possível. O teste foi realizado três vezes em cada braço, alternando-os e com pausas para descanso. Para a análise, foi considerada a FPP máxima obtida no teste.

A força muscular foi dicotomizada em normal e diminuída. A força muscular diminuída foi definida conforme os pontos de corte recomendados pelo *European Working Group on Sarcopenia in Older People* (EWGSOP2), sendo <27 kgf para homens e <16 kgf para mulheres^(4)^.

As variáveis independentes incluíram idade, sexo, número de comorbidades e IMC. O peso foi aferido utilizando uma balança eletrônica digital (Tanita^®^), com precisão de 0,01 grama. A altura foi aferida com um estadiômetro móvel. A presença de morbidades foi mensurada por meio do relato do participante sobre o diagnóstico médico das doenças, por meio da pergunta: “Quais doenças o(a) Sr.(a) já foi diagnosticado(a) pelo médico?”. Foi considerada uma lista de 17 doenças: diabetes, hipertensão, excesso de peso/obesidade, desnutrição, colesterol elevado, triglicerídeos elevados, osteoporose, câncer, acidente vascular cerebral (AVC), infarto do miocárdio, asma, bronquite, Doença Pulmonar Obstrutiva Crônica (DPOC), catarata, enxaqueca, depressão e gastrite.

### Análise dos resultados e estatística

Os dados foram analisados no software STATA, versão 14.0.

Inicialmente, todas as variáveis foram analisadas de forma descritiva, utilizando média, mediana, desvio padrão, frequência absoluta e relativa. Posteriormente, a amostra foi caracterizada com base na média e no desvio padrão (DP).

A validade da força muscular autorreferida (medidas A, B, C e D) foi analisada em relação ao método padrão-ouro (FPP)^(4,20,21)^. A validade foi determinada por meio de cinco características: sensibilidade, especificidade, valores preditivos positivo (VPP) e negativo (VPN), e acurácia.

Para tal, foi elaborada uma tabela 2×2 entre o padrão-ouro e cada medida autorreferida, permitindo o cálculo das seguintes medidas de validade:

1. Sensibilidade: proporção de verdadeiros positivos (positivos pela medida referida e aferida) dividida pela soma de verdadeiros positivos e falsos negativos (positivos pela medida aferida e negativos pela referida);
2. Especificidade: proporção de verdadeiros negativos (negativos pela medida referida e aferida) dividida pela soma de verdadeiros negativos e falsos positivos (negativos pela medida aferida e positivos pela referida);
3. VPP: proporção de verdadeiros positivos dividida pela soma de verdadeiros positivos e falsos positivos;
4. VPN: proporção de verdadeiros negativos dividida pela soma de verdadeiros negativos e falsos negativos; e
5. Acurácia: proporção da soma de verdadeiros positivos e verdadeiros negativos em relação ao total da amostra (N).

## RESULTADOS

A amostra foi composta por 187 idosos da comunidade, sendo 66,8% mulheres com 78,8±5,9 anos. Os indivíduos apresentaram 4,0±2,1 comorbidades e IMC de 27,3±5,3 kg/m^2^. As mulheres apresentaram menor FPP (20,4±5,5 kg) em comparação aos homens (32,7±9,4 kg) (p<0,001) (Tabela 1).

**Tabela 1:** Caracterização da amostra total e segundo sexo, Goiânia, Goiás, Brasil, 2018

Variáveis	Total (N = 187)		Mulheres (n = 125)		Homens (n = 62)		Valor de *p*
	Média	±DP	Média	±DP	Média	±DP	
Idade (anos)	78,8	5,9	78,7	6,1	79,1	5,4	0,092
Multimorbidade	4,0	2,1	4,1	2,2	3,8	2,1	0,294
IMC (kg/m^2^)	27,3	5,3	27,7	5,6	26,5	4,5	0,466
FPP (kg)	24,5	9,1	20,4	5,5	32,7	9,4	**<0,001**

*DP - Desvio Padrão; FPP - Força de Preensão Palmar; IMC - Índice de Massa Corpórea.*

**Tabela 2:** Capacidade preditiva das medidas autorreferidas de força muscular em relação ao padrão-ouro, Goiânia, Goiás, Brasil, 2018

Medidas subjetivas de força	Força de Preensão Palmar (padrão-ouro)				
	Sensibilidade (IC 95%)	Especificidade (IC 95%)	VPP (%)	VPN (%)	Acurácia (%)
Geral					
Medida A	71,7	17,1	79,0	12,2	61,5
Medida B	41,1	41,7	74,7	14,4	41,2
Medida C	31,7	74,2	71,6	14,2	34,8
Medida D	32,5	52,9	75,4	15,0	36,2
Mulheres					
Medida A	71,7	16,7	83,5	9,1	63,7
Medida B	47,2	21,1	76,9	6,7	43,2
Medida C	38,7	31,6	76,9	8,5	37,6
Medida D	36,8	38,9	78,0	9,5	36,8
Homens					
Medida A	71,7	12,5	70,2	13,3	56,5
Medida B	26,7	68,8	70,6	25	37,7
Medida C	15,5	68,8	58,3	22,4	29,5
Medida D	22,2	68,8	66,7	23,9	34,4

*A - redução de força; B - dificuldade de levantar 5kg; C - baixa pontuação SARC-F; D - redução de força + levantar 5kg; IC - Intervalo de Confiança; VPP - Valor preditivo positivo; VPN - Valor preditivo negativo.*

A prevalência força muscular diminuída em idosos variou conforme o método (Figura 1), sendo a menor aquela que tomou como referência a avaliação da FPP.

**Figura 1:** Prevalência de força muscular diminuída em idosos, segundo critérios aferidos e autorreferidos, Goiânia, Goiás, Brasil, 2018

*FPP - Força de Preensão Palmar (aferida e classificada segundo ponto de corte da EWGSOP); Medida A, B, C e D (autorreferidas, conforme diferentes critérios citados na metodologia).*

Em relação a capacidade preditiva das 4 medidas referidas de força, na Tabela 2 observa-se que a diminuição referida da força (Medida A) apresentou maior sensibilidades, VPP e acurácia entre as variáveis analisadas, tendo valores semelhantes entre mulheres e homens. A baixa pontuação no SARC-F (Medida C) apresentou maior especificidade, especialmente entre os homens, contudo teve acurácia inferior quando comparada as outras medidas avaliadas. Destaca-se que todas os métodos subjetivos avaliados apresentaram VPP significativo com exceção da Medida C para o sexo masculino.

## DISCUSSÃO

Buscando contribuir para o planejamento de ações sustentáveis no sistema de saúde e adequadas às demandas dos idosos, este estudo analisou a validade de medidas autorreferidas utilizadas em pesquisas para avaliação da força muscular diminuída, considerando como padrão-ouro a FPP. Buscam-se avaliações com alta sensibilidade e boa especificidade. De modo geral, os resultados mostraram que as medidas A e C investigadas atendem razoavelmente a um ou outro parâmetro, mas não ambos simultaneamente, o que as torna inelegíveis para a afirmação do fenômeno estudado.

Porém, considerando outras propriedades, como a acurácia e o valor preditivo positivo, verifica-se que a medida A apresenta boa acurácia e valor preditivo positivo para as mulheres (83,5% e 63,7%, respectivamente). Para os homens, nenhuma das medidas apresentou acurácia adequada. Esse cenário indica que, na ausência do dinamômetro para avaliação da FPP, as demais medidas devem ser utilizadas em associação, especialmente para os homens, a fim de subsidiar a tomada de decisão sobre as intervenções necessárias pela equipe multiprofissional, na qual a enfermagem pode trazer contribuições significativas.

Adicionalmente, é importante destacar que, na realização de pesquisas na área que envolvam a redução da força muscular como variável de exposição ou desfecho, as medidas autorreferidas devem ser evitadas.

No presente estudo, considerando o resultado obtido com o uso do dinamômetro hidráulico (medida padrão-ouro adotada), a prevalência de baixa força muscular encontrada (18,7%) foi superior à observada na coorte de idosos do estudo SABE^(22)^, no qual foi de 7,5%. No entanto, foi similar aos resultados da coorte ELSI, na qual, utilizando-se os mesmos pontos de corte, encontrou-se uma prevalência de 16,6% entre os homens e 17,7% entre as mulheres^(9)^. Essas divergências devem-se aos diferentes pontos de corte adotados nos estudos mencionados. Tanto a presente investigação quanto o estudo ELSI-Brasil utilizaram os pontos de corte recomendados pelo *European Working Group on Sarcopenia in Older People*, parâmetro amplamente adotado em estudos internacionais, nos quais foram encontradas prevalências similares às deste estudo^(23,24)^.

A baixa força muscular estimada pela medida A (baixa percepção de força) apresentou a maior prevalência (74,2%), comparada às demais. Resultados de estudos que utilizaram essa forma de avaliação da força muscular apresentam ampla variação, como 80,0% em Minas Gerais^(25)^ e 40,7% no Tocantins^(26)^.

A medida B (dificuldade em levantar 5 kg), relatada por 44,7% dos idosos neste estudo, foi referida em estudos anteriores por 27,3% dos idosos coreanos^(27)^ e por 35% dos idosos da Grã-Bretanha^(28)^.

A medida C (pontuação ≥4 no SARC-F), que revelou uma prevalência de 35,5% de baixa força muscular, foi reportada em outros estudos com prevalência de 18,8% em idosos turcos com mais de 65 anos^(29)^, 16,6% em idosos chineses com mais de 65 anos^(30,31)^ e 39,8% em idosos chineses com mais de 70 anos^(30,31)^. No Brasil, a prevalência estimada pela medida C variou amplamente, sendo de 4,5% em idosas com mais de 60 anos da Região Sul^(32)^ e de 23,0% em idosos de ambos os sexos da Região Sudeste^(33)^.

As diferenças de prevalência entre as medidas autorreferidas podem ser atribuídas à sua construção semântica. Sabe-se que medidas subjetivas são passíveis de vieses, uma vez que, para respondê-las, as pessoas interpretam subjetivamente os enunciados. Assim, a pergunta referente à medida A (percepção de diminuição de força nos últimos 12 meses) é mais genérica e, dessa forma, indicaria uma força geral; adicionalmente, remete a um estado dinâmico de evolução, ou seja, o respondente avalia a diminuição da força e não propriamente se ela está reduzida, atingindo um estado aquém do que seria considerado ideal para essa funcionalidade.

As medidas B e C, por incluírem o critério “dificuldade para carregar peso maior que 5 kg”, podem remeter a uma percepção mais específica. Isso pode impactar diretamente na quantidade de casos rastreados como positivos. É importante considerar que, ao estabelecer padrões comparativos, como levantar e carregar 5 kg, seria prudente completar o enunciado da pergunta para aumentar a concretude do construto. Uma possibilidade seria: “Dificuldade de carregar um saco de 5 kg de arroz por 30 segundos?”. O tempo de 30 segundos é coerente com o ponto de corte para o teste de velocidade de marcha^(4)^. Esse acréscimo poderia aprimorar a definição do cenário de avaliação e evitar interpretações diversificadas, que podem comprometer as medidas comparativas dentro de uma mesma população.

É oportuno destacar que essas duas medidas têm sido apresentadas com apenas duas opções de resposta. Para estudos futuros, sugere-se a inclusão das opções: “nenhuma”, “pouca”, “moderada” e “grande”, permitindo investigar se diferentes pontos de corte apresentam melhor sensibilidade, especificidade, VPN, VPP e acurácia.

O instrumento proposto por Nunes et al.^(19)^ foi originalmente desenvolvido para a detecção da síndrome de fragilidade em idosos, com base no fenótipo de fragilidade^(8)^. Naquele contexto, o item referente à força apresentou boa sensibilidade (77,7%) para fragilidade, mas, por outro lado, especificidade de 34,9%, VPP de 44,7% e VPN de 69,8%^(19)^. Como o item referente à perda de força do instrumento autorreferido apresentou boa carga fatorial e consistência interna^(19)^, ele foi considerado um possível instrumento para a avaliação exclusiva da redução de força. Essa medida tem alta capacidade de selecionar verdadeiros positivos e, mesmo que não exclua muito bem os verdadeiros negativos, é um instrumento promissor para o rastreamento de idosos, possibilitando futuras avaliações e intervenções relacionadas à redução da força^(34,35)^.

A medida B apresentou bons valores de VPP tanto na amostra geral quanto na estratificada por sexo, além de uma especificidade de 68,8% para os homens. Não foram encontrados outros estudos que demonstrassem a sensibilidade, especificidade, VPP, VPN e acurácia especificamente do item de força presente no SARC-F. Isso provavelmente ocorre devido à utilização de um padrão de comparação mais específico para que os idosos avaliem se atingem ou não esse critério, conforme mencionado anteriormente.

Com relação à medida C (baixa pontuação no SARC-F), estudos internacionais relataram variações nos valores de sensibilidade, que oscilaram entre 15% e 40,3%, e de especificidade, que ficaram entre 82% e 99%, para ambos os sexos^(27-29,36)^.

Na Região Sul do Brasil, encontrou-se uma sensibilidade de 58,9% e especificidade de 82,1% (14) para a medida C. Valores semelhantes foram observados na Região Sudeste, com sensibilidade de 60,0% e especificidade de 80,92%^(37)^.

Quando a amostra é estratificada por sexo, observa-se uma importante diminuição da especificidade da medida C (baixa pontuação no SARC-F) entre as mulheres. Resultados similares foram encontrados em um estudo chinês^(38)^ e outro coreano^(27)^. No Brasil, não foram identificados estudos dessa natureza.

Devido às numerosas complicações associadas à redução da força muscular, tais como aumento da mortalidade, hospitalização, quedas e diminuição da independência e autonomia dos idosos^(5-7)^, a detecção precoce dessa condição é fundamental para orientar ações em saúde, principalmente no contexto da Atenção Primária à Saúde (APS)^(19,39)^.

### Limitações do estudo

A pesquisa envolveu como participantes usuários da rede de atenção básica à saúde, o que pode representar um fator limitante para a reprodutibilidade da estimativa da prevalência da força muscular diminuída em idosos da população geral.

Outro fator limitante refere-se a potenciais fatores de confusão que não foram incluídos na análise de validade dessas medidas, tais como condições agudas, inflamatórias e osteomusculares, que podem comprometer a mensuração da força muscular em idosos e, consequentemente, a validade das medidas. Apesar disso, as multimorbidades dos idosos foram analisadas por meio de uma lista de 17 condições crônicas, as quais são frequentes e recomendadas para uso em estudos com esse grupo.

Contudo, para o objetivo maior da investigação, que foi estimar a sensibilidade, especificidade, valor preditivo positivo e negativo, e acurácia de medidas autorreferidas da força muscular, os achados do estudo ainda se mostram relevantes.

### Contribuições para a área da enfermagem e saúde pública

Tendo em vista a busca pela promoção do envelhecimento saudável da população idosa, com a otimização das capacidades funcionais ao longo dos anos, a incorporação do monitoramento da força muscular pelo enfermeiro na APS possibilita a identificação precoce de alterações funcionais^(39)^, subsidiando ações preventivas e de reabilitação para idosos atendidos na APS.

Diante disso, este estudo evidenciou que as medidas autorreferidas A (percepção de redução de força muscular) e C (baixa pontuação no SARC-F) mostram-se úteis para incorporação à prática clínica da enfermagem, podendo ser adicionadas à consulta de enfermagem para avaliação geriátrica ampla (AGA).

Além disso, quando incorporado às avaliações de rotina, assim como a verificação de sinais vitais e do IMC, o uso de um teste autorreferido para avaliação da força muscular pode servir como uma ferramenta de triagem para sarcopenia e fragilidade, sendo de fácil aplicação e baixo custo.

A avaliação da força muscular também oferece uma oportunidade para a realização de educação em saúde sobre medidas que promovem sua preservação, como a prática regular de atividade física, especialmente exercícios de fortalecimento muscular. Além disso, possibilita a orientação sobre os riscos de quedas para idosos e seus cuidadores.

É importante contextualizar que, embora o foco da presente investigação seja a população idosa, a força muscular reduzida é um fator relacionado (fator causal/coadjuvante) ou uma característica definidora (evidência) em quatorze diferentes diagnósticos de enfermagem na taxonomia NANDA-I^(40)^. Isso reforça a aplicabilidade dos resultados desta pesquisa em outros cenários da prática e a necessidade de estudos futuros para a validação de diagnósticos de enfermagem.

Considerando que a pergunta sobre “levantar e carregar 5 kg” integra as questões da *Caderneta de Saúde da Pessoa Idosa*, proposta pelo Ministério da Saúde^(41)^, é fundamental ressaltar que os resultados desta investigação servem como um alerta para os profissionais de saúde. Não se deve utilizar esse item como único parâmetro para o julgamento da presença de força muscular reduzida. Dessa forma, o estudo contribui para a prática baseada em evidências nos serviços de atenção básica à saúde de idosos, no que se refere à avaliação da força muscular.

A promoção do envelhecimento saudável da população idosa envolve a otimização das habilidades funcionais ao longo do curso da vida. Nesse sentido, a força muscular é uma das medidas que indicam alterações na mobilidade e que devem ser monitoradas no contexto da APS^(39)^.

A verificação da força muscular em idosos na prática de enfermagem auxilia na identificação precoce da diminuição da força, uma condição associada a problemas como fragilidade, quedas, perda de independência e maior risco de hospitalizações.

Este estudo evidenciou que as medidas autorreferidas A (percepção de redução de força muscular) e C (baixa pontuação no SARC-F) são úteis para incorporação à prática clínica da enfermagem, seja na consulta de enfermagem, no exame físico dos idosos ou em campanhas de rastreamento. Além disso, essas medidas podem subsidiar o planejamento de cuidados específicos ou interprofissionais, com a participação de familiares, a curto, médio e longo prazo, por se tratarem de tecnologias de fácil aplicação.

É oportuno contextualizar que, embora o foco da presente investigação seja a população idosa, a força muscular reduzida configura-se como um fator relacionado (fator causal/coadjuvante) ou uma característica definidora (evidência) em quatorze diferentes diagnósticos de enfermagem na taxonomia NANDA-I^(40)^. Isso aponta para a aplicabilidade dos resultados desta pesquisa em outros cenários da prática, além da necessidade de estudos futuros para a validação de diagnósticos de enfermagem.

Considerando que a pergunta sobre “levantar e carregar 5 kg” integra as questões presentes na *Caderneta de Saúde da Pessoa Idosa*, proposta pelo Ministério da Saúde^(41)^, é importante ressaltar que os resultados desta investigação devem servir de alerta aos profissionais para que não utilizem esse item como único parâmetro para o julgamento da presença de força muscular reduzida. Dessa forma, este estudo contribui para a prática baseada em evidências dos profissionais de saúde nos serviços de atenção básica à saúde dos idosos, no que se refere à avaliação da força muscular.

## CONCLUSÕES

Para um diagnóstico confiável e um valor preditivo adequado da força muscular diminuída, é necessário o uso de medidas válidas, como a FPP, além de alternativas em caso de ausência do dinamômetro. Nesse sentido, as medidas A e C mostram-se mais adequadas para a triagem da perda de força entre idosos na APS.

A medida A, por apresentar grande capacidade de selecionar indivíduos para o rastreamento, e a medida C, pela capacidade de excluir falsos positivos, podem, em conjunto, indicar um grupo prioritário para intervenção. Contudo, as medidas não se mostraram suficientemente adequadas para recomendação como ferramenta de triagem da redução de força em idosos da comunidade nos serviços de saúde. No entanto, considerando sua fácil aplicabilidade na prática clínica e profissional, a adoção desses parâmetros pode ser útil para discriminar idosos que podem necessitar de intervenções preventivas para mitigar os desfechos adversos de saúde associados à perda de força muscular.

## Data Availability

Os dados da pesquisa estão disponíveis no corpo do artigo.
